# Convergent evolution of quadrupedality in ornithischian dinosaurs was achieved through disparate forelimb muscle mechanics

**DOI:** 10.1098/rspb.2022.2435

**Published:** 2023-02-08

**Authors:** Matthew Dempsey, Susannah C. R. Maidment, Brandon P. Hedrick, Karl T. Bates

**Affiliations:** ^1^ Department of Musculoskeletal & Ageing Science, Institute of Life Course & Medical Sciences, University of Liverpool, The William Henry Duncan Building, 6 West Derby Street, Liverpool L7 8TX, UK; ^2^ Department of Earth Sciences, The Natural History Museum, Cromwell Road, London SW7 5BD, UK; ^3^ Department of Biomedical Sciences, College of Veterinary Medicine, Cornell University, 930 Campus Road, Ithaca, NY 14853, USA

**Keywords:** macroevolution, biomechanics, multi-body dynamics, dinosaurs, quadrupedality, moment arms

## Abstract

The secondary evolution of quadrupedality from bipedal ancestry is a rare evolutionary transition in tetrapods yet occurred convergently at least three times within ornithischian dinosaurs. Despite convergently evolving quadrupedal gait, ornithischians exhibited variable anatomy, particularly in the forelimbs, which underwent a major functional change from assisting in foraging and feeding in bipeds to becoming principal weight-bearing components of the locomotor system in quadrupeds. Here, we use three-dimensional multi-body dynamics models to demonstrate quantitatively that different quadrupedal ornithischian clades evolved distinct forelimb musculature, particularly around the shoulder. We find that major differences in glenohumeral abduction–adduction and long axis rotation muscle leverages were key drivers of mechanical disparity, thereby refuting previous hypotheses about functional convergence in major clades. Elbow muscle leverages were also disparate across the major ornithischian lineages, although high elbow extension muscle leverages were convergent between most quadrupeds. Unlike in ornithischian hind limbs, where differences are more closely tied to functional similarity than phylogenetic relatedness, mechanical disparity in ornithischian forelimbs appears to have been shaped primarily by phylogenetic constraints. Differences in ancestral bipedal taxa within each clade may have resulted in disparate ecomorphological constraints on the evolutionary pathways driving divergence in their quadrupedal descendants.

## 1. Introduction

**Figure 1.**
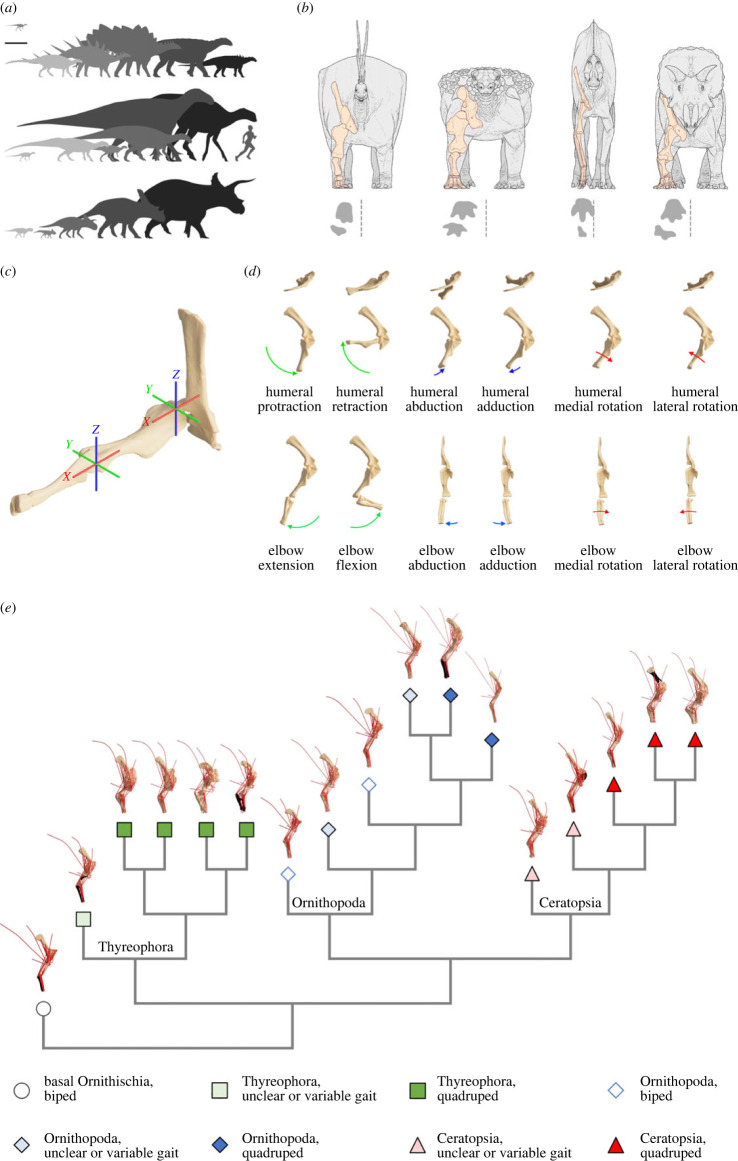
(*a*) Ornithischian bauplan disparity represented by silhouettes of the taxa from this study in lateral view (scaled to the main reference specimens). Silhouettes are: (top row) *Lesothosaurus* and thyreophorans, (middle row) ornithopods, (bottom row) ceratopsians. Left–right order within clades as in (*e*). Scale bar = 1 m. (*b*) Hypothetical postures of major quadrupedal clades in anterior view, with the scapulocoracoid and forelimb skeleton highlighted and generalized trackway placements relative to the midline (e.g. [49–52]). (Left–right) Stegosauria, Ankylosauria, Hadrosauria, Ceratopsidae. Not to scale. (*c*) Model reference pose with all joint angles at 0° demonstrated by *Tenontosaurus*, with joint axes highlighted. (*d*) Joint actions demonstrated by *Tenontosaurus* in dorsal and lateral views (glenohumeral actions, elbow extension and flexion), and anterior view (elbow abduction–adduction and elbow mediolateral long axis rotation). (*e*) Simplified phylogenetic relationships of the taxa in this study, displayed with the musculoskeletal models of each in lateral view. Models as shown in (*f*) are posed at 20° glenohumeral protraction, 90° glenohumeral adduction, 0° glenohumeral long axis rotation, 45° elbow flexion and 0° elbow abduction– adduction and long axis rotation. From left to right: *Lesothosaurus*, *Scelidosaurus*, *Kentrosaurus*, *Stegosaurus*, *Peloroplites*, *Animantarx*, *Hypsilophodon*, *Tenontosaurus*, *Dysalotosaurus*, *Mantellisaurus*, *Iguanodon*, *Brachylophosaurus*, *Psittacosaurus*, *Protoceratops*, *Avaceratops*, *Chasmosaurus*, *Triceratops*. Bones rendered in silhouette represent schematically sculpted areas for which scans were not available (see electronic supplementary material, §S1, table S2). Not to scale.

Deciphering the functional or biomechanical implications of morphological change, as well as phylogenetic constraints on that change, are key components to understanding major adaptive radiations in the fossil record. Biomechanical assessments can, for example, shed light on how anatomical innovations enabled major behavioural niche adaptations over geological time (e.g. [1]) and how patterns of anatomical change may be influenced (or limited) by physical constraints on biological form (e.g. [2]). The secondary evolution of quadrupedality from bipedal ancestors is rare in tetrapods. Reversion to quadrupedality is only known to have occurred in ornithodiran archosaurs, most notably occurring multiple times within Dinosauria and closely related outgroups (e.g. Silesauridae) [3,4]. In dinosaurs, only herbivorous taxa evolved quadrupedality, and this occurred convergently in the long-necked sauropodomorphs, and at least three times in ornithischians, including in the armoured thyreophorans, the duck-billed hadrosauriform ornithopods, and the horned ceratopsians (figure 1*a,b,e*) [3]. Each of these groups was able to attain massive body size (figure 1*a*), and on each occasion, the reversion to quadrupedality resulted in major radiations of both morphological and ecological diversity that shaped the composition of terrestrial ecosystems throughout the Mesozoic era [5].

The forelimbs of ornithischian dinosaurs underwent a profound functional change during the evolution of quadrupedality, switching from being used in a range of behaviours (e.g. foraging, feeding and digging) in early diverging bipeds [6,7] to become a fundamental part of the weight-bearing locomotor apparatus in quadrupeds. Previous studies have identified common osteological correlates to quadrupedality across Ornithischia but demonstrated that quadrupedal characters were not constrained to occur in a specific order of acquisition [8,9]. This suggests that the selective pressures driving quadrupedal evolution may have been varied across clades, and that quadrupedal locomotor styles may have been disparate [8,9]. Other studies have demonstrated that despite acquiring similar quadrupedal features, body shapes and limb proportions were highly disparate both within and between major ornithischian clades, leading to inferences of postural divergence [10–12]. This divergence might be expected to require or benefit from different forelimb musculoskeletal mechanics. Extant quadrupedal animals show adaptations in the arrangements of their forelimb muscles associated with different modes of locomotion (e.g. [13–17]), suggesting that basic measures of muscle function can also guide interpretation of hypotheses regarding overall limb mechanics in extinct taxa. In bipedal archosaurs, quantitative studies of muscle leverage in fossil and extant taxa have provided insights into convergent musculoskeletal evolution in archosaur hind limbs [18,19], and shifts in the mechanisms of bipedal gait along the archosaurian lineage to birds [20].

Despite the potential of these quantitative approaches, and debate surrounding forelimb mechanics in specific clades (e.g. [10,21–24]), previous assessments of changes to forelimb muscle mechanics during the evolution of quadrupedality across Ornithischia as a whole have been entirely qualitative. Maidment & Barrett [8] qualitatively reconstructed forelimb myology across Ornithischia and subsequently inferred both convergent and disparate mechanisms of forelimb control and locomotor posture (figure 1*a,b*). Therein, they suggested that stegosaurs and ceratopsids convergently evolved similar methods of glenohumeral adduction and mediolateral rotation to maintain a splayed ‘press-up’ stance, with hadrosaurs, by contrast, employing predominantly glenohumeral abduction muscle leverages to maintain a narrow-gauge stance. Ankylosaurs, despite their close phylogenetic relationship to stegosaurs, were proposed to have maintained a distinctive wide-gauge stance via splayed distal forearms, with the humerus maintaining a vertical orientation controlled by abductor muscle leverage [8].

Herein, we apply multi-body dynamics analysis (e.g. [15,17–20,25–33]) to a sample of ornithischian forelimbs representing the wide array of bauplans present throughout the clade (figure 1*a,e*) to quantitatively investigate, for the first time to our knowledge, how basic forelimb muscle mechanics and function changed across the ornithischian tree, and the extent to which the clades that evolved quadrupedal posture also exhibited mechanical convergence in their solutions to quadrupedal locomotion.

## 2. Material and methods

### (a) Musculoskeletal model construction

Musculoskeletal reconstructions were created for 17 ornithischian taxa, representing an array of bauplans from most of the major clades (figure 1*a,e*; electronic supplementary material, §S1, figures S7–S23). Models of the right forelimb and pectoral girdle of each taxon were constructed based on three-dimensional scans of fossil material generated via multiple methods, including photogrammetry, laser scanning, and computed tomographic (CT) scanning. Specimen details are outlined in electronic supplementary material, §S1, table S2. Fossils with complete forelimbs were selected where possible, although some models required reconstruction based on multiple individuals, mirroring of left-side elements, or modelling of missing elements informed by records of other specimens and related taxa (electronic supplementary material, §S1, table S2). Available models of complete skeletons, as well as photographs, measurements, and skeletal reconstructions from the literature were used to estimate the torso proportions of each modelled taxon to inform the attachments and pathway shapes of axially originating musculature (electronic supplementary material, §S1, table S2).

Model construction followed a similar approach to that of previous studies (e.g. [29], see also electronic supplementary material, §S1). Model articulation and joint centre estimation were carried out via a circle fitting method, with manual translation accounting for unpreserved cartilaginous epiphyses and ensuring consistency with ornithischian forelimbs preserved in articulation (see electronic supplementary material, §S1). The relative joint axis orientations and rotational centres (figure 1*c,d*) followed the approach of previous studies of tetrapod forelimbs (e.g. [17,30,34], see also electronic supplementary material, §S1), in which each joint was modelled with three rotational axes passing through fixed centres. For the glenohumeral joint, the axis parented to the scapulocoracoid and oriented to the long axis of the glenoid is referred to as abduction–adduction, the axis parented to the humerus and oriented to its long axis is referred to as mediolateral long axis rotation, and the axis that passes through the humeral head perpendicular to both the abduction–adduction and mediolateral long axis rotation axes is referred to as protraction–retraction (figure 1*c,d*). For the elbow joint, the axis parented to the humerus that passes through both distal humeral epicondyles is referred to as extension–flexion, the axis parented to the antebrachium and oriented to its long axis is referred to as mediolateral long axis rotation, and the axis passing through the lateral distal humeral epicondyle perpendicular to both the extension–flexion and mediolateral long-axis rotation axes is referred to as abduction–adduction (figure 1*c,d*).

Models were initially constructed in a standardized reference pose from which 0° at all joint angles was defined, based on previous studies (e.g. [17,30]) (figure 1*c*). These were subsequently placed into a standardized range of functional poses that fell within the likely range of possible weight-bearing poses for the quadrupedal taxa, and from which muscle moment arms were extracted (see also electronic supplementary material, §S1). For the glenohumeral joint, the standardized functional poses were defined at 5° intervals between 40° humeral retraction and 40° humeral protraction, with the humerus adducted by 90° (the distal epicondyles pointing ventrally), neither medially nor laterally rotated, and with the elbow at 45° flexion (figure 1*d*). For the elbow joint, the functional poses were defined at 5° intervals between 10° and 100° elbow flexion, in which the humerus is adducted by 90°, protracted by 20°, and neither medially nor laterally rotated (figure 1*d*).

Twenty-three forelimb muscles were reconstructed using osteological correlates for muscle attachment based on the archosaur and sauropsid extant phylogenetic bracket (EPB) [35], incorporating information from previous studies (e.g. [8,36–40], see also electronic supplementary material, §S1). Muscles were modelled as pathways extending from origin to insertion in the musculoskeletal multi-body dynamics software GaitSym2017 (https://github.com/wol101/GaitSym_2017). Sixteen of the 23 muscles were interpreted to have had broad attachment sites (electronic supplementary material, §S1, table S3). In these cases, three strands were modelled, representing the estimated posterior, anterior and midline pathways. The general approach to muscle pathway setup was to reduce subjective decision making in the reconstruction of muscle shapes by using minimal constraints to allow each strand to reach its insertion while preserving the relative musculoskeletal layering expected from the EPB, ensuring that, where possible, muscle pathway differences primarily reflected the widely disparate morphologies of the modelled taxa. This was achieved by using wrapping cylinders, which are geometrically simplified regions through which a given muscle pathway must not pass. Model articulation, joint and muscle setups, and both the advantages and disadvantages of wrapping cylinder use are discussed in further detail in electronic supplementary material, §S1. The moment arms of each muscle were calculated across the standardized range of postures using the muscle–tendon unit travel path method by processing the raw GaitSym data outputs in MATLAB (http://www.mathworks.com) and normalizing by minimum humeral shaft circumference. Bone circumference has been shown to be a stronger postcranial proxy for overall body mass in tetrapods than bone length [41,42], and was therefore chosen as the size-normalization metric. To facilitate comparisons across taxa, we calculated the minimum, maximum and mean moment arms for each muscle for each joint function across our postural range. For multi-strand muscles, the mean, maximum and minimum were calculated from the values obtained from all strands. As mostly similar qualitative patterns were found between the major clades across different metrics (electronic supplementary material, §§S2–S8; see also electronic supplementary material, §S1 for additional discussion), here we focus on mean moment arms, and present the mean protraction–retraction, abduction–adduction and mediolateral long axis rotation moment arms of each glenohumeral muscle, and the mean extension–flexion, abduction–adduction and mediolateral long axis rotation moment arms of each elbow muscle.

### (b) Sensitivity analysis

We repeated analysis of the glenohumeral muscle moment arms for representative taxa from each major quadrupedal group (*Stegosaurus*, *Peloroplites*, *Brachylophosaurus*, *Chasmosaurus*) across three variant model sets to test for the effect of stance width and scapula orientation on relative moment arm patterns (electronic supplementary material, §S1, figures S24–S43, and §S9). To quantify the effects of stance width we generated two additional model iterations, one in which the humeri were placed in a splayed orientation (75° adduction), and the second in a tucked orientation (115° adduction) (electronic supplementary material, S1, figure S24). To examine the effect of scapular orientation, we generated an additional model iteration in which the scapular slope was increased to 70° (electronic supplementary material, §S1, figure S25), which is consistent with steeper scapular orientations suggested for multiple dinosaur groups [43,44]. Across the three glenohumeral sensitivity variant sets, the qualitative order of the summed moment arms and moment arm ratios remained consistent, and the major qualitative patterns between taxa did not overlap (electronic supplementary material, §S1, figures S26–S43). Only two specific instances of changes in the qualitative ordering of taxa within sets were observed, occurring in the more adducted variant set (electronic supplementary material, §S1, figures S30–S33). As key hypotheses on postural inference from glenohumeral moment arms are underpinned by inter-clade differences (which are preserved in our sensitivity analyses) we conclude that, while moment arm magnitudes vary quantitively with abduction–adduction and scapular slopes, major qualitative differences between the ornithischian clades are unaffected.

To assess whether cartilaginous structures of the elbow that may differ in shape from the underlying osteology affected interpretations (e.g. [34]), we also generated two variant model sets of the same quadrupedal taxa, in which the position of the elbow abduction–adduction rotational centre was mediolaterally translated from the position in the base model set, in which the axis passes through the middle of the lateral distal epicondyle of the humerus, to one-third and two-thirds across the width of the condyle, respectively (electronic supplementary material, §S1, figures S44–S47; see also electronic supplementary material, §S9). We found that the qualitative ordering of summed elbow abduction–adduction moment arms remained consistent in each variant set, but that the moment arms quantitatively varied (the more lateral the position of the joint axis, the greater the abduction moment arms, and *vice versa* for adduction) (electronic supplementary material, §S1, figures S45 andS46). This quantitative variation led to overlap in the range of results from the variant sets, with the exception of elbow adduction moment arms in *Chasmosaurus*, the range of which remained above that of the other representative taxa (electronic supplementary material, §S1, figure S46). While the actual position of the elbow abduction-adduction joint axis may be uncertain in fossil archosaurs, we conclude that as long as the placement of the joint axis is consistent, then the qualitative ordering of the summed moment arms should remain unchanged.

### (c) Data analysis

**Figure 2.**
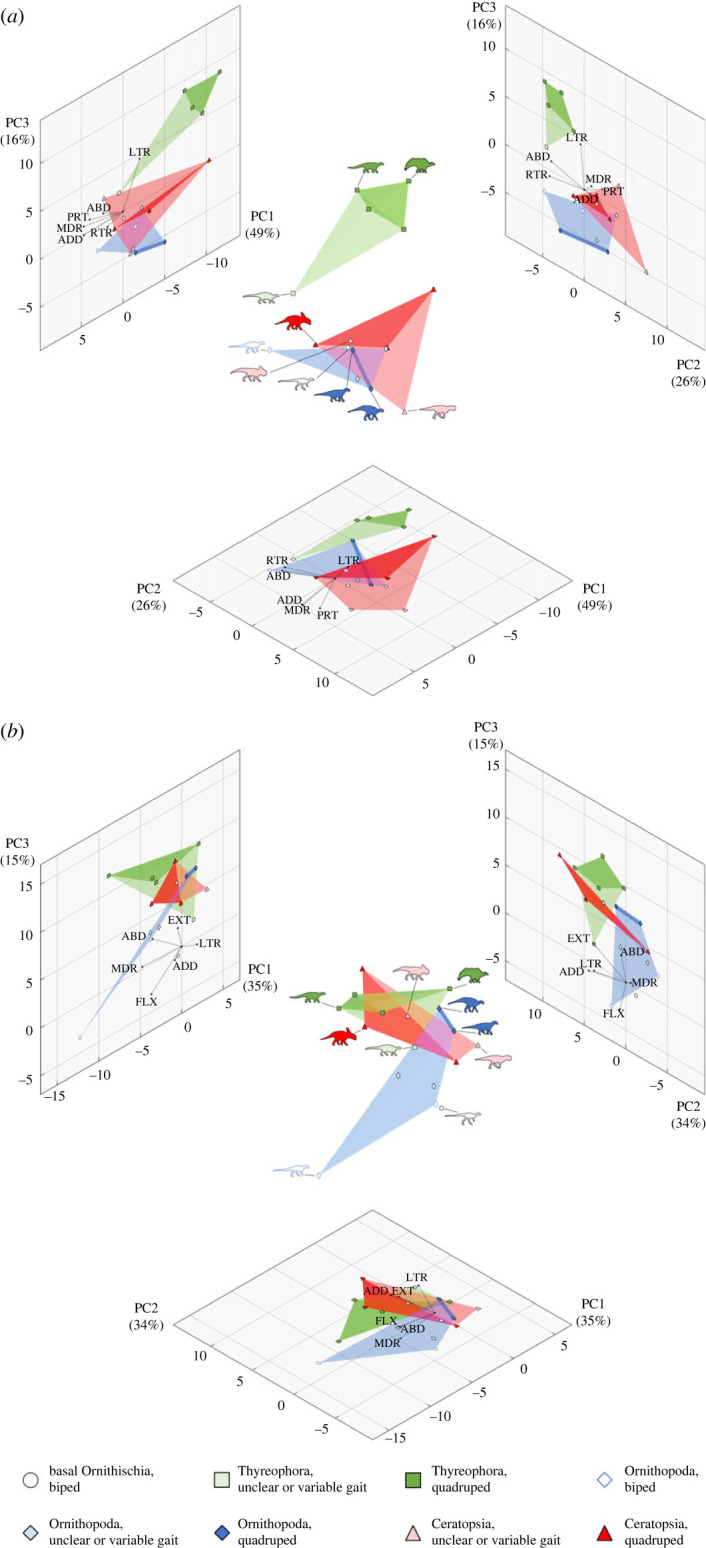
Three-dimensional morphospaces for phylogenetic principal components analysis (pPCA) analyses of summed moment arm magnitudes, glenohumeral musculature (*a*) and elbow musculature (*b*). In (*a*), PRT = glenohumeral protraction, RTR = glenohumeral retraction, ABD = glenohumeral abduction, ADD = glenohumeral adduction, MDR = glenohumeral medial long axis rotation, and LTR = glenohumeral lateral long axis rotation. In (*b*), EXT = elbow extension, FLX = elbow flexion, ABD = elbow abduction, ADD = elbow adduction, MDR = elbow medial long axis rotation, and LTR = elbow lateral long axis rotation. Taxa highlighted in (*a*) are, clockwise from top: *Peloroplites*, *Stegosaurus*, *Psittacosaurus*, *Brachylophosaurus*, *Iguanodon*, *Lesothosaurus*, *Protoceratops*, *Hypsilophodon*, *Chasmosaurus*, *Scelidosaurus*. Taxa highlighted in (*b*) are clockwise from top: *Protoceratops*, *Stegosaurus*, *Brachylophosaurus*, *Iguanodon*, *Psittacosaurus*, *Lesothosaurus*, *Hypsilophodon*, *Scelidosaurus*, *Chasmosaurus*, *Peloroplites*. Loading vectors displayed at 500% for clarity.

We calculated the summed glenohumeral protraction, retraction, abduction, adduction, medial rotation and lateral rotation moment arm magnitudes, as well as the summed elbow extension, flexion, abduction, adduction, medial rotation and lateral rotation magnitudes from the mean moment arms. The summed moment arms calculated from the mean values are treated herein as a simplified general proxy for the overall differences in forelimb muscle leverages, as opposed to representations of more complex *in vivo* biomechanical metrics. To examine the relationship between multiple moment arms for multiple functions simultaneously, we used phylogenetic principal components analysis (pPCA) [45] via the *phytools* package (v. 1.0-1) [46] for R, where the evolutionary correlation matrix was derived assuming a Brownian motion model of trait evolution. This allowed us to assess the collective differences in moment arms for all functions across major ornithischian clades (figure 2; electronic supplementary material, §S10). The overall phylogenetic relationships of the taxa included in our study are well established, and details of tree topology and potentially phylogenetically labile taxa are discussed in the electronic supplementary material (electronic supplementary material, §S1, text and figure S48). Branch lengths were calculated using the timePaleoPhy() function in the *paleotree* package (v. 3.4.4) [47] for R, using 1.0 Myr minimum branch lengths. Additionally, a second set of pPCA morphospaces were generated from a tree with uniform branch lengths, and a third set of morphospaces were generated from a principal component analysis (PCA) with no phylogenetic input via prcomp() in R (electronic supplementary material, §S1, figures S49–S52, and §S10). Only the pPCA morphospace with 1 Myr branch lengths is presented here, as similar patterns were found in all three morphospace sets, qualitatively suggesting that the most major patterns of divergence between taxa were driven primarily by functionally derived differences in the summed muscle moment arms.

## 3. Results

### (a) Summed muscle moment arms and morphospaces

PCs1–3 explain 91% of the variation in the data when only glenohumeral moment arms are included in the pPCA (figure 2*a*), 84% when only elbow moment arms are included (figure 2*b*), and 74% when all summed muscle moment arms are included (electronic supplementary material, §S1, figure S53, and §S10).

In the glenohumeral muscle moment arm morphospace (figure 2*a*), PC1 (representing 49% of the variation) is strongly positively correlated with summed glenohumeral medial rotation, adduction and protraction moment arms. PC2 (representing 26% of the variation) is strongly negatively correlated with summed glenohumeral retraction and abduction moment arms. PC3 (representing 16% of the variation) is strongly positively correlated with summed glenohumeral lateral rotation moment arms. The closely related ornithopods and ceratopsians show overlap in glenohumeral muscle moment arm morphospace, with the three-dimensional convex hulls centred around the early-diverging bipedal ornithischian *Lesothosaurus* (figure 2*a*). The quadrupedal ceratopsids and hadrosauriform ornithopods remain segregated on PC3 owing to higher glenohumeral lateral rotation moment arms in ceratopsids (figure 2*a*). The early-diverging ceratopsian *Psittacosaurus* was found to be separated from other ceratopsians on PC2 and PC3 in the glenohumeral muscle moment arm morphospace (figure 2*a*) as a result of lower summed abduction and lateral rotation moment arms. While the non-ceratopsid neoceratopsian *Protoceratops* clustered more closely with the ceratopsids in the glenohumeral muscle moment arm morphospace compared with *Psittacosaurus*, high summed glenohumeral protraction moment arms still drove some separation (figure 2*a*). The stegosaurs and ankylosaurs are highly divergent from all other taxa in the glenohumeral muscle moment arm morphospace, resulting mainly from relatively low summed glenohumeral adduction moment arms, and relatively high summed glenohumeral abduction and lateral rotation moment arms (figure 2*a*). By contrast, the early-diverging thyreophoran *Scelidosaurus* clustered more closely with the other ornithischian clades (figure 2*a*). Overall, the relative distribution of taxa from the three main ornithischian lineages that evolved quadrupedality (Thyreophora, Ornithopoda and Ceratopsia) is strongly divergent in the glenohumeral moment arm morphospace.

In the elbow muscle moment arm morphospace (figure 2*b*), PC1 (representing 35% of the variation) is strongly negatively correlated with summed elbow medial rotation, flexion and abduction moment arms. PC2 (representing 34% of the variation) is strongly positively correlated with elbow adduction, lateral rotation and extension moment arms. PC3 (representing 15% of the variation) is moderately positively correlated with summed elbow abduction moment arms, and moderately negatively correlated with summed elbow flexion moment arms. The overall distribution of the main ornithischian lineages shows mixed patterns of convergence and divergence in elbow muscle moment arms, both between and within clades, although each primarily quadrupedal group is still separated in three-dimensional morphospace when PCs1, 2 and 3 are all considered. Earlier-diverging ornithischians from each major clade are spread widely across the morphospace, but obligate bipedal taxa are separated from most primarily quadrupedal taxa on PC3 as a result of higher elbow flexion moment arms and lower elbow extension moment arms. High elbow extension and adduction moment arms drive high PC2 scores in the stegosaur *Kentrosaurus*, the ankylosaurs, and the chasmosaurine ceratopsids, whereas the stegosaur *Stegosaurus* and the centrosaurine ceratopsid *Avaceratops* have low or negative PC2 scores as the result of lower elbow adduction (*Stegosaurus*) and lower elbow adduction and extension (*Avaceratops*) moment arms than their close relatives. The quadrupedal ornithopod *Iguanodon* has a similarly negative PC2 score as the result of low elbow extension and adduction moment arms. The ankylosaur *Peloroplites* is strongly separated from other quadrupedal taxa on PC1 owing to higher elbow medial rotation moment arms.

The major differences identified in the summed moment arm pPCA morphospaces are also qualitatively recovered when the ratios of the summed muscle moment arms are compared (electronic supplementary material, §S1, figures S54–S71), indicating that key patterns are not a product of normalization by humeral circumference.

### (b) Individual muscle functions

The full array of moment arm data extracted from each individual muscle is provided in electronic supplementary material, §§S2–S8. Herein, we focus on key individual muscles that underpinned the differences in the summed moment arm values driving the morphospace disparity (figure 2), or otherwise stood out as exhibiting noteworthy differences between the major ornithischian clades.

In most taxa, m. deltoideus clavicularis (DCL) was recovered with mixed protraction–retraction moment arms (electronic supplementary material, §§S2 and S3). However, DCL was recovered with retraction moment arms across more of the postural range in ankylosaurs than in other taxa, resulting in a mean retraction moment arm (electronic supplementary material, §§S2 and S3). By contrast, DCL was recovered with almost entirely protraction moment arms in *Brachylophosaurus*, resulting in a higher mean protraction arm than in any other taxon (electronic supplementary material, §§S2 and S3). Reduced DCL protraction moment arms in ankylosaurs are congruent with the pattern shown by the summed protraction moment arms and protraction/retraction ratios, which were lower in the ankylosaurs than in other quadrupedal taxa (electronic supplementary material, §S1, figures S54 and S56). The high summed glenohumeral protraction moment arms and high protraction/retraction ratios of *Protoceratops* (electronic supplementary material, §S1, figures S54 and S56) appear to mostly result from high mean protraction moment arms in m. biceps brachii (BBR) (electronic supplementary material, §S2 and §S3).

**Figure 3.**
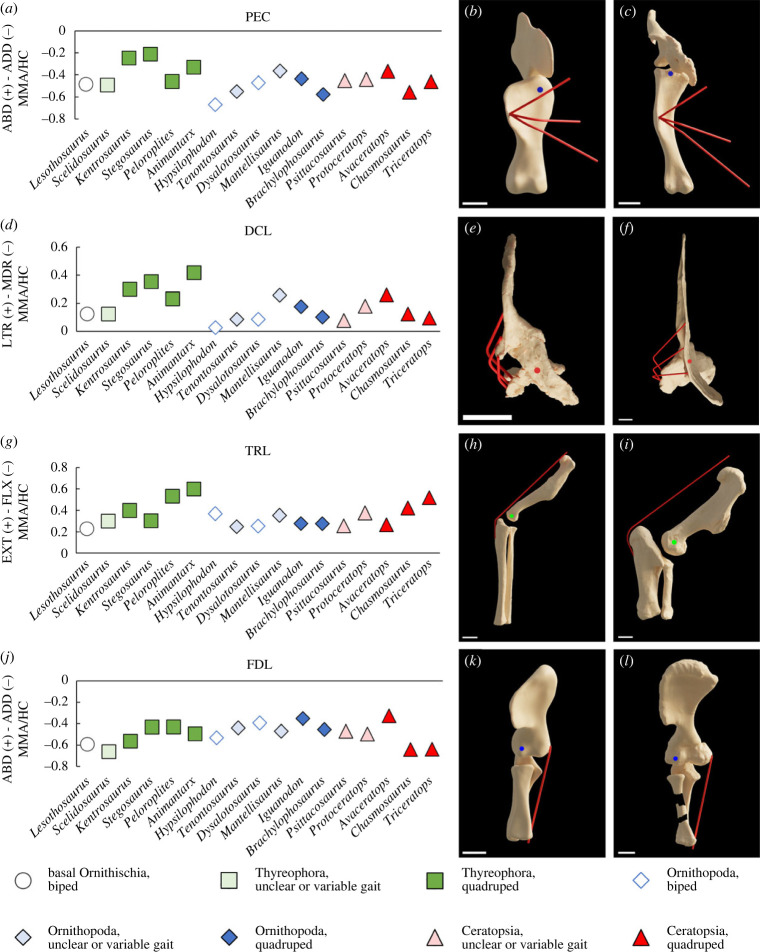
Mean moment arms measured across different joint actions for key muscles in all taxa, normalized to humeral circumference. M. pectoralis (PEC) glenohumeral abduction–adduction (*a*), PEC viewed antero-ventrally down the aspect of the aspect of the abduction–adduction axis in *Stegosaurus* (*b*) and *Brachylophosaurus* (*c*); m. deltoideus clavicularis (DCL) glenohumeral mediolateral long axis rotation (*d*), DCL viewed dorsally down the aspect of the long axis rotation joint axis in *Animantarx* (*e*) and *Triceratops* (*f*); TRL) elbow extension (*g*), TRL viewed laterally down the aspect of the extension–flexion axis in *Brachylophosaurus* (*h*) and *Triceratops* (*i*); m. flexor digitorum longus (FDL) elbow adduction (*j*), FDL viewed anteriorly down the aspect of the abduction-adduction joint axis in *Stegosaurus* (*k*) and *Chasmosaurus* (*l*). The circle in each model render represents the joint axis, with colours as in figure 1*c,d*. Scale bars = 0.1 m. In each glenohumeral render, models are posed at 0° glenohumeral protraction–retraction, 90° glenohumeral abduction–adduction and 0° degrees glenohumeral long axis rotation. In each elbow render, models are posed at 45° elbow extension–flexion, 0° elbow abduction–adduction, and 0° elbow mediolateral long axis rotation.

M. pectoralis (PEC) is a key driver of differences in summed glenohumeral abduction arms, exhibiting a lower mean adduction moment arm across stegosaurs than in other taxa (figure 3*a*). By contrast, the mean adduction moment arms of PEC were greater in *Brachylophosaurus* than any other quadrupedal taxon (figure 3*a*), followed closely *Chasmosaurus*. This is congruent with the pattern shown by the summed adduction moment arms, which were higher in *Brachylophosaurus* and *Chasmosaurus* than in other quadrupedal taxa (electronic supplementary material, §S1, figure S58).

DCL is a key driver of differences in summed glenohumeral lateral rotation moment arms, with greater mean lateral rotation moment arms in the quadrupedal thyreophorans than in most other taxa (figure 3*d*). The glenohumeral long axis rotation moment arms of m. latissimus dorsi (LAT) also differ between clades, with moderately high mean lateral rotation arms in the thyreophorans and ceratopsids as well as numerous other earlier-diverging ornithischian taxa, but low mean lateral rotation moment arms in the hadrosauriform ornithopods, with the mean switching to glenohumeral medial rotation in *Mantellisaurus* (electronic supplementary material, §S5).

Most quadrupedal ornithischians had greater summed elbow extensor moment arms than bipedal ornithischians (electronic supplementary material, §S1, figure S63), contributing to the separation of bipedal and quadrupedal taxa in the elbow muscle morphospace, particularly across PC2 and PC3. Mean elbow extension moment arms were highest in m. triceps longus (TRL) and m. triceps brevis (TRB) in all taxa (electronic supplementary material, §S6). Mean elbow extension moment arms in TRL and TRB were also higher in the ankylosaurs and chasmosaurine ceratopsids than most other taxa (figure 3*g*).

Muscles spanning the lateral surface of the antebrachium are abductors of the elbow, whereas muscles spanning the medial surface of the antebrachium are adductors of the elbow. Muscles spanning the elbow joint more anteriorly or posteriorly (e.g. TRL, BBR) were mostly found to have lower elbow abduction–adduction moment arms, as their primary function is elbow extension and flexion. The moment arms of most elbow adductors exceeded those of adductors (electronic supplementary material, §S1, figures S66–S68, §S2 and §S7). Key elbow adductors such as m. flexor digitorum longus (FDL) were found to have particularly high moment arms in the chasmosaurine ceratopsids (figure 3*j*).

In all modelled taxa, the majority of muscles spanning the antebrachium had low long axis rotation moment arms (electronic supplementary material, §S1, figures S69 andS70, §S2 and §S8), although the mean lateral rotation moment arm of m. epitrochleoanconeus exceeded that of other muscles. M. anconeus was also found to have greater medial rotation moment arms in most taxa when the elbow was positioned in more flexed postures, and had a relatively high mean medial rotation moment arm in both the ankylosaurs and chasmosaurine ceratopsids (electronic supplementary material, §S2 and §S8).

**Figure 4.**
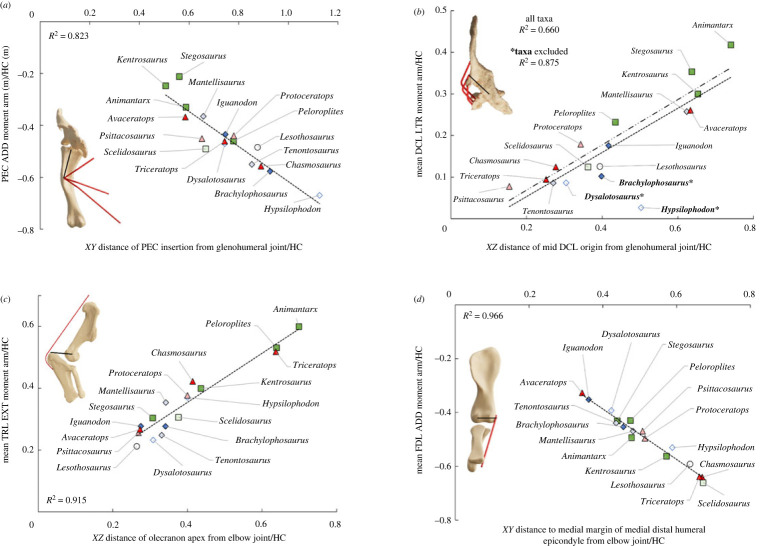
Osteological measurements plotted against mean moment arm magnitudes for key muscles measured across different joint actions for key muscles in all taxa, normalized to humeral circumference. M. pectoralis (PEC) glenohumeral abduction–adduction plotted against the distance of the PEC insertion from the glenohumeral joint centre in the *XY* plane of the humerus (*a*); m. deltoideus clavicularis (DCL) glenohumeral long axis rotation plotted against the distance of the DCL midpoint origin from the glenohumeral joint centre in the *XZ* plane of the scapulocoracoid (*b*); m. triceps longus (TRL) elbow extension–flexion plotted against the distance of the olecranon apex from the elbow joint centre in the *XZ* plane of the antebrachium (*c*); m. flexor digitorum longus (FDL) elbow abduction–adduction plotted against the distance of the medial margin of the medial distal humeral epicondyle from the elbow joint centre in the *XY* plane of the humerus (*d*). Example model render inserts are *Brachylophosaurus* (*a*), *Animantarx* (*b*), *Triceratops* (*c*) and *Stegosaurus* (*d*)*,* and are viewed down the aspects of the relevant segments, with the black lines labelling each highlighted osteological measurement. The dash–dot trendline in (*b*) excludes taxa marked with * owing to the centres of their acromial ridges/processes being placed anterior to the glenohumeral joint rather than posterior as in all other taxa. Olecranon proportions in *Lesothosaurus*, *Scelidosaurus*, *Animantarx* and *Iguanodon* (*c*) are estimated from reconstructed antebrachia (see electronic supplementary material, §S1, table S2).

Muscle moment arms representative of major mechanical disparities between taxa showed very strong correlations with the size or displacement of homologous osteological features measured from the rotational centres of the joint axis in planar view (figure 4; electronic supplementary material, §S11). The mean glenohumeral adduction moment arm of PEC strongly correlated with the laterodistal displacement of its insertion on the apex of the deltopectoral crest (figure 4*a*), and the mean glenohumeral lateral rotation moment arm of DCL strongly correlated with the posterolateral displacement of its central origin on the acromial process (figure 4*b*). The mean elbow extension moment arm of TRL (and TRB) strongly correlated with the elongation and posterior expansion of the olecranon process (figure 4*c*), and the elbow adduction moment arm of FDL strongly correlated with the expansion of the medial distal epicondyle of the humerus (figure 4*d*). The strength of these correlations demonstrates that the major patterns in the data (figures 2 and 3) were driven primarily by observable osteological differences (figure 4), and are therefore not the product of the methods used to represent muscles in the models.

## 4. Discussion

Convergently acquired osteological correlates have previously been shown to characterize the evolution of quadrupedality in each ornithischian lineage [8,9]. Qualitative assessments of muscle leverage also suggested some common or convergent patterns of shoulder muscle evolution [8]. By contrast, our analyses strongly emphasize clade-specific quantitative differences in shoulder muscle moment arms in quadrupedal ornithischians, providing clear evidence that there was no universal constraint for a specific mechanical arrangement of muscles about the glenohumeral joint as forelimb function shifted to facilitate weight support and locomotion in Ornithischia. The segregation of quadrupedal ornithischian clades in the pPCA shoulder muscle morphospace (figure 2*a*) emphasizes that each individual group underwent a reorganization of pectoral musculature during their evolution of quadrupedality. This contrasts with quadrupedal ornithischian hind limbs, in which moment arm patterns in pelvic musculature were previously suggested to be more constrained by functional convergence than phylogenetic relatedness [26]. For example, patterns such as an increase in hip abduction moment arms were highlighted as occurring across the convergent switch to a more columnar stance in quadrupedal taxa [26]. Unlike the hind limbs, the disparity in quadrupedal ornithischian forelimb muscle leverage may have been the result of differing forelimb functions (e.g. digging, food retrieval) in early-diverging bipedal members of each clade. This is reflected by our data, as early-diverging taxa from different clades do not cluster particularly tightly in morphospace, particularly in elbow muscle moment arm morphospace (figure 2), and therefore were likely to have had functionally disparate forelimbs. As forelimbs were co-opted for locomotion during the evolution of each major ornithischian clade, pathways to quadrupedality were perhaps different owing to constraints imposed by ancestral function.

Muscles responsible for glenohumeral abduction–adduction and long-axis rotation appear to be key drivers of increased mechanical disparity (figures 2 and 3). Overall, this contrasts with previous qualitative inferences of shoulder muscle evolution, which postulated a mixture of divergence and convergence in shoulder muscle leverage across the ornithischian lineages [8]. For example, based on the morphology of the pectoral girdle, Maidment & Barrett [8] hypothesized that the moment arms of PEC, DCL and DSC would have been similar in stegosaurs and ceratopsids. However, our musculoskeletal models indicate that the shoulder musculature of stegosaurs was mechanically different from ceratopsids, and comparable to the more closely related ankylosaurs (figures 2 and 3). Stegosaurs had lower summed adduction and medial rotation moment arms, but higher lateral rotation moment arms than ceratopsids (figures 2 and 3; electronic supplementary material, §S1, figures S57–S62). This was further reflected in key individual muscles. PEC was found to have higher mean adduction moment arms in chasmosaurine ceratopsids than in stegosaurs, with ankylosaurs and the smaller centrosaurine ceratopsid *Avaceratops* falling in between (figure 3*a*). High PEC adduction moment arms in chasmosaurine ceratopsids appear to be driven mostly by a distal and lateral displacement of its insertion from the glenohumeral joint, as a result of the deltopectoral crest becoming both more distally displaced and laterally broader (figure 4*a*). DCL was recovered with higher mean lateral rotation moment arms in the stegosaurs and the ankylosaurs than in the chasmosaurine ceratopsids. DCL mean lateral rotation moment arms in *Avaceratops* slightly exceeded the ankylosaur *Peloroplites*, but still fell below the other quadrupedal thyreophorans (figure 3*d*). Higher DCL lateral rotation moment arms appear to be driven by greater posterolateral displacement of the acromial process from which DCL originates (figures 3*e* and 4*b*).

Maidment & Barrett [8] hypothesized that despite osteological differences, the moment arms of key pectoral muscles in hadrosaurs did not differ greatly from early-diverging bipedal taxa. We found some quantitative support for this hypothesis, as *Brachylophosaurus* and several other ornithopods clustered relatively closely around *Lesothosaurus* on either PC1 or PC3 in the glenohumeral muscle moment arm morphospace. However, ornithopods were spread more broadly overall, particularly across PC2 (figure 2*a*), corresponding to variation in abduction and retraction moment arms. *Iguanodon* was separated from other primarily or facultatively quadrupedal ornithopods (figure 2*a*) by lower summed glenohumeral adduction and higher summed glenohumeral abduction moment arms (electronic supplementary material, §S1, figures S57 and S58). While *Iguanodon* bears qualitative anatomical similarities to related taxa, its forelimbs are proportionally distinct and more massively constructed [48], and may have been subject to considerably different mechanical demands during stance and locomotion.

Maidment & Barrett [8] hypothesized that the pectoral musculature of hadrosaurs (specifically DCL and DSC) provided high abduction moment arms to control the collapse of the forelimb into adduction, facilitating the narrow-gauge stance suggested by trackways [49], which contrasts with the wide-gauge stance of quadrupedal thyreophorans and ceratopsids [50–52]. However, we find little support for high abduction moment arms in hadrosaurs, as *Brachylophosaurus* showed lower abduction moment arms than most quadrupedal taxa (electronic supplementary material, §S1, figure S57). *Brachylophosaurus* also had the second highest summed adduction moment arms of any quadrupedal taxon, exceeded only slightly by *Chasmosaurus* (electronic supplementary material, §S1, figure S58), driven particularly by PEC as a result of the deltopectoral crest being strongly distally displaced from the glenohumeral joint (figures 3*c* and 4*a*). While high glenohumeral adduction moment arms have been correlated with wider-gauge postures in extant quadrupeds (e.g. [15,17]), our findings suggest caution is needed in their use as indicators of more abducted limb postures in quadrupedal animals more generally.

At least three factors may explain (from a functional perspective) the absence of the expected elevated glenohumeral abduction moment arms in most ornithopods. First, selection for high glenohumeral abduction moment arms was generally lower relative to other functions in ornithopods than other quadrupedal ornithischians, perhaps related to more parasagittal limb motions and greater locomotor performance in line with their more streamlined body forms and cursorial limb proportions [11]. Second, the manus may have been typically placed directly beneath as opposed to medial to the shoulder joint, and therefore the shoulder experienced both adduction and abduction ground reaction force moments during habitual gaits as limbs were in stance phase. Third, retention of a plesiomorphic more posteriorly positioned centre of mass [12] led to different gait dynamics (e.g. different loading between the forelimbs and hind limbs) in ornithopods, and subsequently placed different mechanical demands on their forelimb muscles relative to other quadrupedal ornithischians.

Elbow muscle moment arms were found to have mixed patterns of convergence and divergence between and within the different ornithischian lineages (figure 2*b*). Elbow extension moment arms (figure 3*g*; electronic supplementary material, §S1, figure S63) and extension/flexion ratios (electronic supplementary material, §S1, figure S65) were higher in most quadrupedal taxa, largely owing to the triceps tendon becoming posteriorly displaced by the elongation and posterior expansion of the olecranon process of the ulna (figures 3*hi* and 4*c*). Increased elbow extension moment arms, therefore, represent a common convergent feature among most quadrupedal ornithischians, but nevertheless still show systematic variation between taxa. In particular, greater elbow extension moment arms in the triceps are recovered in the ankylosaurs and chasmosaurine ceratopsids, and summed elbow extension moment arms were highest in *Triceratops*. Ceratopsids in general may have had a more anterior centre of mass relative to other ornithischians [12], and the largest taxa possess more robust humeri than smaller taxa [11], and thus may have supported greater weight on their forelimbs. Large extant quadrupeds in which the forelimb takes on an increased role in weight-bearing also have high triceps leverages, which similarly result from an enlarged olecranon [16]. Increased elbow extension moment arms in the triceps have also been identified in sauropods with an enlarged olecranon [31].

High elbow adductor moment arms have been shown to correlate with more sprawled postures in extant taxa [13], and result from the medial displacement of muscles originating from the medial distal humeral epicondyle (figures 3*k–l* and 4*d*). Ceratopsid dinosaurs have often been reconstructed with more splayed elbows than other quadrupedal ornithischians (e.g. [21–24]), which may be supported by our recovery of high elbow adduction moment arms in *Chasmosaurus* and *Triceratops*, in which the medial distal humeral epicondyle is greatly enlarged (figure 3*j*; electronic supplementary material, §S1, figure S67). The non-ceratopsid neoceratopsian *Protoceratops*, suggested in previous works to have been able to facultatively vary its stance (e.g. [9,53]), was also recovered with high summed elbow adduction moment arms, which is consistent with prior inferences of a splayed quadrupedal forelimb posture [13]. However, elbow adduction moment arms were found to be low in the centrosaurine ceratopsid *Avaceratops*. The degree to which the elbows were habitually splayed may therefore have varied between ceratopsians, although owing to the more ventral orientation of the glenoid than in extant sprawling reptiles, it is unlikely that they exhibited a completely sprawling gait. High elbow moment arms were also not unique to ceratopsians, and were found in other quadrupedal taxa such as the stegosaur *Kentrosaurus*, as well as earlier-diverging taxa such as *Scelidosaurus* and *Hypsilophodon* (figure 3*j*; electronic supplementary material, §S1, figure S67).

Specific predictions of postures are difficult to test from muscle moment arms alone [19,27], but the differences we recover between the different major quadrupedal ornithischian clades suggest that if they did employ similar joint kinematics, then they must have done so with varying levels of mechanical efficiency. While previous qualitative interpretations of forelimb muscle mechanics have focused on snapshots of stance phase posture [8], it is equally possible that the patterns of muscle moment arm evolution recovered here represent mechanical adaptations for swing phase kinematics. For example, the relatively high glenohumeral lateral rotation and abduction moment arms of quadrupedal thyreophorans may have assisted swing phase motion in these wide-bodied animals. Evaluating such hypotheses about three-dimensional limb motion and *in vivo* biomechanical metrics is now technologically feasible using dynamic models and gait optimization approaches (e.g. [28,33]). The reconstruction of three-dimensional musculoskeletal arrangements and the determination of muscle moment arms are foundational to the deployment of these dynamic gait simulations; however, a major hurdle to their effectiveness lies in deriving values for muscle architecture and contractile properties [54,55].

## 5. Conclusion

Our study is the first to our knowledge to quantify modifications to the three-dimensional arrangement of forelimb musculature across each major ornithischian clade as they independently evolved quadrupedality (figure 1). Our results emphasize that thyreophorans, ornithopods and ceratopsians each evolved quadrupedality through different patterns of rearrangement of musculature around the shoulder and elbow joint (figures 2–4). While we find support for some prior qualitative inferences of muscle evolution (e.g. high glenohumeral medial rotation moment arms in ceratopsids), our quantitative data refute the hypotheses of strong convergence between some major clades and therefore favour a model of mechanical divergence across Ornithischia as a whole. The phylogenetic disparity in the mechanical arrangement of shoulder musculature is consistent with the differences in limb proportions [11] and overall body shape [12] seen in each quadrupedal group. This contrasts with quadrupedal ornithischian pelvic muscle mechanics, which appear to vary more according to function than to phylogenetic constraints. An increase in elbow extensor muscle leverage appears to be a convergent hallmark of quadrupedality in heavily built ornithischians (e.g. ankylosaurs and the largest ceratopsids), but even this was differentially expressed across the major lineages according to the variation in body proportions and the resultant increased role of the forelimbs in bearing weight. Mechanical disparity between the weight-bearing forelimbs of the different quadrupedal ornithischians may ultimately have been influenced by functional differences already present in ancestral taxa within each clade, resulting in a disparate set of ecomorphological constraints being placed on their evolutionary pathways.

## Data Availability

Our muscle moment arm and multivariate input–output data are provided in the electronic supplementary material. Model files are available at the following figshare and datacat repositories: https://doi.org/10.6084/m9.figshare.21674723.v2 and https://doi.org/10.17638/datacat.liverpool.ac.uk%2F1711 [56]. The data are provided in the electronic supplementary material [57].
